# Navigating concurrent public health emergencies: Indigenous perspectives from the Cedar Project in British Columbia

**DOI:** 10.1371/journal.pgph.0004658

**Published:** 2025-06-02

**Authors:** Chenoa Cassidy-Matthews, Jorden Hendry, Margo Pearce, Sherri Pooyak, David Zamar, Jeff Reading, Nadine Caron, Martin Schechter, Patricia Spittal, Wayne Christian

**Affiliations:** 1 School of Population and Public Health, Faculty of Medicine, University of British Columbia, Vancouver, British Columbia, Canada; 2 BC Children’s Hospital Research Institute, Vancouver, British Columbia, Canada; 3 The Cedar Project Partnership, Kamloops, British Columbia, Canada; 4 Faculty of Health Sciences, Simon Fraser University, Burnaby, British Columbia, Canada; PLOS: Public Library of Science, UNITED STATES OF AMERICA

## Abstract

Connection and resilience are critical to the health and wellbeing of Indigenous Peoples who use drugs (IPWUD): connection to family, cultural supports, safer coping mechanisms and circles of care. Urban IPWUD are more likely to face multiple harms from emergency public health restrictions alongside ongoing toxic drug and housing crises in British Columbia. This paper aims to amplify the experiences of urban IPWUD as they navigated the COVID-19 pandemic and the corresponding public health response in Vancouver and Prince George, BC. Nineteen semi-structured interviews were completed with Indigenous Peoples enrolled in the Cedar Project COVID-19 Study in Vancouver (n = 9) and Prince George (n = 10). Interpretive description was adapted to identify themes across participants’ stories. Emerging themes were brought back to participants for member checking using iterative techniques. Interviews were conducted in person between March-May 2022. The median age of participants was 36, with 37% men and 63% women. Four themes were identified: 1) Enduring impacts of colonialism worsened pandemic-related stress for Indigenous Peoples, led to 2) Cycles of isolation, uncertainty and crisis destabilized Indigenous Peoples, and 3) Fear and Trauma-Driven Distrust amplified experiences of grief and loss amidst conflicting public health protocols. However, 4) Resilience and connection were important buffers against pandemic-related harm. Urban IPWUD face ongoing public health emergencies that threaten daily security, safety, and health. This study demonstrated the complex ways Indigenous People navigated COVID-19 and concurrent emergencies, and the challenges they faced. These findings shed light on the ongoing discrimination against urban IPWUD, and reiterate that they are the experts in their own needs and determining how to best survive and thrive through health emergencies.

## Introduction

Indigenous People who use drugs (IPWUD) in British Columbia (BC) are facing concurrent public health emergencies: COVID-19, the toxic drug supply crisis, and the housing crisis. It is likely that each creates additional risk for the other. Substance use may increase COVID-19 risk through sharing smoking/injection equipment, barriers to physical distancing and personal hygiene, and housing instability [1–3]. COVID-19 and its response (e.g., isolation protocols) have increased the risk of violence, affected mental well-being, and exacerbated substance use-related harms (e.g., overdose due to isolated drug use) [4,5] while impeding access to sources of wellness such as family and cultural connection. The curtailing of harm reduction resources coupled with safe supply shortages in urban areas may have also exacerbated substance use-related harms like sexually transmitted and blood-borne infections.

### Settler colonialism

To understand COVID-19 experiences among Indigenous Peoples, we must first consider the impact of colonialism. The history and impacts of legislated genocide by colonial European settlers in what is now called Canada, and British Columbia in particular, has been extensively documented; the Indian Act proclaimed in 1876 enabled the federal Canadian government jurisdiction over Indigenous lives, lands, and futures. The Residential School system was the primary tool for assimilating Indigenous Peoples into western Euro-centric culture by way of forcible removal of children from families to attend boarding schools which were aimed at “removing the Indian from the child” by stripping them of all elements of their respective cultures and forbidding them from speaking their language [6]). The first residential school opened in the late 1860s and the last one closed in 1996, with peak genocidal operations occurring between 1879–1946 [7].

Today, we navigate structurally oppressive systems that continue to cause harm through racism and the erosion of cultural buffers that traditionally helped us cope with stress and mediate risk [8–10]. Younger Indigenous People may turn to substance use to cope with the stress of cumulative intergenerational and lifetime traumas, together referred to as complex trauma [11,12]. Cedar and others have demonstrated complex intersections of colonial trauma, substance use, mental health, and resilience, and identified associations affirming that cultural connection is linked with resilience and lower psychological distress [12–14]. Complex trauma passes through generations of Indigenous families and manifests in health challenges including higher chance for HIV/HCV transmission [15], and we expected that complex trauma would also introduce greater chance of exposure to COVID-19, greater barriers to vaccination, and that its impacts may be substantial among people already navigating trauma. But how does this complex trauma come to put us in a position of vulnerability to COVID-19 and barriers to vaccination?

### Indigenous survivance

During uncertain times, Indigenous *survivance*, the combination of survival and resistance [16] and historical resilience [17], requires purpose, connection, and resilience, defined as the ability to cope with changes and uncertainty, and withstand trauma to carry on with a meaningful life [18,19]. Having a sense of purpose helps people to stay positive through times of uncertainty [20] and encourages stronger social networks and resilience to stress [21,22]. For example, Indigenous Peoples who have a strong sense of community belonging reported better overall mental health [23]. Connection in any form is critical to the health and well-being of IPWUD: connection to family, cultural supports, and circles of care. Survivance*,* more than describing survival, celebrates survival through the mobilization of both traditional and contemporary knowledge in response to present-day problems. Stories of Indigenous survivance are uncommon in the western healthcare literature, which limits our ability as researchers to envision outcomes beyond mere survival; and limits the visibility and recognition of Indigenous Peoples’ strengths and resilience during health emergencies. Thus, this study aimed to understand, through the lens of survivance, the impact of concurrent public health emergencies during COVID-19 on Indigenous People who use drugs in Vancouver and Prince George, British Columbia.

## Methods

### Ethics statement

At the Cedar Project, we take a relational ethics approach in our research. Cedar participants are cherished relations whose stories are honoured, and wellbeing is sacred. A critical component of the Cedar Project is to ensure that site offices are culturally safe, welcoming settings without judgment of drug use, where police are not welcomed, and Indigenous identity is honoured. All participants are offered support through referrals to care, access to harm reduction supplies and education, and access to safe, quiet spaces to rest during the day. The Cedar Project research team represents a collaboration of Indigenous and allied investigators. The Cedar Project COVID-19 Study was granted ethics approval from the UBC-Providence Health Care Research Institute Ethics Board (REB certificate number: H20-03229).

### The Cedar Project

This work builds upon established research infrastructure and existing trust-based relationships with over 800 Indigenous People who use drugs (IPWUD) in Vancouver and Prince George in the Cedar Project. The Cedar Project is an ongoing CIHR-funded cohort study that monitors the health and wellbeing of IPWUD in British Columbia who are between the ages of 15–50. Beginning in 2003, the Cedar Project has semi-annually assessed demographic characteristics, historical and lifetime traumatic events, drug-use patterns, and sexual health among participants through a standard survey. As health priorities emerge internally from findings from the standard survey, and externally through public health priorities, policy changes, and emergencies, additional surveys and in-depth interview guides are developed in addition to the standard survey. The primary site offices in Prince George and Vancouver, BC have remained unchanged since the study’s inception. Indigenous collaborators and investigators, collectively known as the Cedar Project Partnership, govern the research process.

### Methodology

Indigenous Knowledge of pandemics and emergencies calls upon ancient wisdom mobilized in contemporary circumstances, and the coauthors of this study—comprised of 60% Indigenous researchers including myself (C.M.) as a mixed European settler and anishininewak member of Sachigo Lake First Nation—employed teachings from our own lives to the methods and interpretations of findings presented here. IPWUD’ stories are honoured in this study through relational epistemology, by collaboration in interpreting the findings and paying special attention to (w)holistic descriptions of *what* participants experienced and *how* they experienced it to best inform future pandemic responses.

### Recruitment & data collection

Indigenous Peoples who use or have used substances were enrolled to the Cedar Project COVID-19 Study between 01-01-2021 and 31-12-2022. I conducted 19 semi-structured interviews in Vancouver (n = 9) and Prince George (n = 10) using the conversational method [24] between 01-03-2022 and 31-05-2022. Initial written and ongoing verbal informed consent was obtained with each participant. The median age of participants was 36 years, with 37% men and 63% women. Participants were recruited through purposive sampling methods, which involved identifying participants with diverse experiences and identities in each study site. With the aid of a topic guide developed by the primary author (C.M.), co-authors (M.P., S.P.), and senior authors (P.S.), our interviews explored concepts selected a priori including: the impact of the pandemic from the beginning; strategies used to stay safe; navigating COVID-19 and quarantine/self-isolate policies; vaccine perceptions; changing housing circumstances; and experiences of discrimination, racism, violence, and policing. Interview topic guides include strengths-based questions related to secondary impacts of COVID-19 and the pandemic response, asking participants to share how they have navigated the shifting policy environment, mental health and substance use to take care of themselves and loved ones. Interviews were audio recorded and professionally transcribed, and reviewed by interviewer and primary author (C.M.) for accuracy and to remove any remaining identifying information.

### Data analysis

Our analysis used methods adapted from interpretive description (ID) [25] for its explicit emphasis on informing applied practice while allowing room for honouring stories of survival and uplifting Indigenous voices [24–26]. ID has been used in Indigenous health research in Aotearoa New Zealand and Canada, and in community-based participatory research and in harm reduction research [27–29]. I extracted data in whole-story form, which refers to a method of data extraction which, unlike line-by-line coding or other forms of fragmentation, data is extracted as a whole thought, experience, or story, and can sometimes run a full page in length. This is widely considered an important characteristic of Indigenous data and aligns with methods of storytelling [24,30–32]. These data stories were stored in a spreadsheet and initially screened using an iterative stepwise technique that followed the “Sticky Notes” method, documented by Burgess and colleagues (2021), who have also used the sticky notes method in combination with ID [28]. Stories were synthesized using sticky notes by noting a short-form reference to the story on a sticky note (since the full story usually would not fit). Sticky notes are a low-barrier analytic tool that can be used at all stages of qualitative research, from data collection, fragmentation and/or whole-story extraction, and reintegration, to analysis, thematic coding, and concept mapping [33–36].

### Stepwise analysis using “sticky notes”

I reviewed the first eight transcripts from Vancouver (n = 4) and Prince George (n = 4) in full and extracted the data in whole-story form into a spreadsheet and then referenced on sticky notes organized by category. Conducting a mid-point analysis was helpful in ensuring that we were still asking the right questions and gave us an opportunity to elicit feedback and analytic support from participants during member-checking before continuing with the analysis. This process followed iterations of the same steps until patterns came together. Categories were chosen according to Burgess et al. (2021) and included “exact words”, “quotable quotes”, “expected but missing”, “possible connections”, “memos”, “concepts” and “sub-concepts” [28]. “Exact words” are the transcript data pulled in original, or close-to whole-story form; “quotable quotes” are exemplary excerpts that demonstrate themes or major patterns for inclusion in manuscripts [25]; “expected but missing” are ideas or concepts that were expected to appear or appear more prominently in the interview data, but did not for known and unknown reasons; “possible connections” are descriptive ideas about the relationships between data and/or concepts; “memos” are researcher notes about feelings, bias, hypotheses, or confusion with the data in parsing the “intellectual chaos” [25]; “concepts” and “sub-concepts” are precursors to themes and are early articulations of underlying patterns emerging in the data. New sticky notes were added as ideas developed, and this process was conducted over multiple iterations until finally six broad concepts were defined. A key difference between the methodological approach documented by Burgess and colleagues [28] and the protocol that we developed for this analysis is the decision to pull data in “whole-story form” rather than “data fragmentation” which aligns with Indigenous Knowledge that posits that stories only keep their meaning in whole form—to fragment stories is to remove their meaning.

### Member-checking

Member-checking is a process whereby essential relationships are confirmed, clarified, and elaborated on through interview and participatory analysis mechanisms, widely used in qualitative research to assess credibility, as an indication of trustworthiness of study findings [25,37,38]. We invited six participants in the Cedar Project who had participated in the original interviews to take part in a *participatory analysis* with one or two members of the research team, including myself and second author (J.H.). Participants were not required to have methodological or technical expertise to participate in the analysis, and we adapted the sticky notes method to include mixed materials like construction paper, posterboard, and coloured markers to help visualize concepts and connections. The connections that emerged from this process and the positive feedback we received from participants suggested this process enabled meaningful engagement in qualitative analysis, a process which frequently excludes research participants. The member-checking process provided key insights that confirmed initial themes, prompted revisions to concepts that didn’t align with participants experiences, and led to the consolidation of overlapping ideas. These refinements directly shaped the final transcript analysis, ensuring it accurately reflected participant perspectives.

## Results

### Overview of findings

Four themes were identified through an iterative analysis involving participants, described in detail below: 1) Enduring impacts of colonialism worsened pandemic-related stress for Indigenous Peoples. This led to: 2) Cycles of isolation, uncertainty and crisis which destabilized Indigenous Peoples; and 3) Fear and Trauma-Driven Distrust amplified experiences of grief and loss amidst conflicting public health protocols. However, 4) Resilience and connection were important buffers against pandemic-related harm.

## Core themes

### Enduring impacts of colonialism worsened pandemic-related stress for Indigenous Peoples

The COVID-19 pandemic reinforced structural and systemic inequity and sanctioned colonial violence. Cedar participants were, directly and indirectly, forced to rely more heavily on survival skills as well as adapt to new circumstances. The pandemic amplified stress associated with precarity, defined in this study as uncertainty coupled with restricted access to resources and support. Colonial responses–by provincial and federal governments–to COVID-19 have not aligned with the needs of urban IPWUD, forcing people into crisis situations:

*For me, COVID’s been like really fucked up. Like um, I dunno it’s hard finding a job, right—it’s hard like, with…prices like I said going up, you know my rent’s pretty high and you know, just to get by and stuff like I find myself stealing…and you know not having money to go out and stuff right. So just being home all the time.* – 43F, Vancouver

#### Financial security.

Financial instability greatly impacted folks relying on welfare and disability assistance, those living with HIV or other chronic health concerns, and people with a criminal record trying to find stable work. These circumstances forced participants to turn to riskier means to make ends meet, like stealing and street security—a casual term participants used to describe cash jobs guarding hide-outs, drug supplies, or other illicit goods for others. For those engaged in sex work, COVID-19 introduced health risks and restrictions that meant fewer and less frequent clients, resulting in diminished income and a few bad dates. Participants discussed the loss of access to resources, services, and support for daily survival. The unpredictability of day-to-day living when the pandemic first began in March 2020 was highlighted frequently by participants as a period of high stress and instability, in which they were unsure what directions to follow.

“COVID money” was a double-edged sword and an example of the insufficient efforts by all levels of government to support constituents where we needed it most: financial security. Increased precarity forced folks to resort to risker means to make ends meet. Many did not feel the amount, nor the handling of the Canadian Emergency Response Benefit (CERB), was appropriate or particularly helpful, especially when other heavily funded causes dominated news cycles, such as the “Freedom Convoy” in 2022, a right-wing protest against vaccine mandates that began on a Facebook page, for which the organizers received unprecedented financial support from multiple individuals and corporations across the country [39,40]. Participants felt this inadequate financial support was a representation of how they are valued by their broader community members and political leaders, and evidence of structural racism during the pandemic [40].

*So, and I mean, that’s what I don’t understand with the COVID thing and that they could have done so many things by reaching out online to get people’s help. And without them having to come out and risk their health, right? They could have still fucking given some money or, you know, like, how, to, to help fund people getting a place to live while this thing was going on. Right? I mean, look at how much money they got for the [Freedom Convoy] truckers. Well, why don’t people that don’t have a place to live and are a little bit at risk, why don’t we count as well? Like, we’re people. We’re somebody’s daughter, we’re somebody’s brother, father, mother, you know?* – 42F, Prince George

Increased financial instability also forced people to change patterns of drug use to meet other basic needs, like food: “I smoke crack once in a while but not as much as I used to. I used to smoke it every day but now I smoke it…when I have money. I don’t, like…like I said, I can’t afford food, so how can I…you know what I mean?” (43F, Vancouver). For participants on safe supply—legal and regulated supply of drugs with mind and body altering properties, that aims to replace drugs accessed through the illicit drug market—most were required to make frequent or daily trips to the pharmacy to renew prescriptions and receive their carries (doses of safe supply that patients are allowed to take home, as opposed to being required to ingest on-site while supervised by a pharmacist or other physician) or to have witnessed ingestion (ingestion of drug dose while under supervision by a pharmacist or physician). With COVID-19 risks and restrictions, this arduous task became more complicated and less accessible, particularly for folks in Prince George in the winter months. All participants who were on safe supply at the time of this study regularly topped up their doses with street supply during COVID-19 to meet their needs.

### Violence and discrimination

Prejudice and discrimination have been a regular experience for those living in the downtown areas of Vancouver and Prince George, but participants felt the severity of these encounters increased during the pandemic. Specifically, acts of discrimination, racism, and violence perpetrated by police officers were discussed. Participants’ experiences ranged from discriminatory behaviour to verbal and physical violence. Participants regularly received glares from non-Indigenous commuters passing by; drivers yelling violent threats out of car windows as they passed by: “people being told they should be dead” (42F, Prince George); racist comments from fellow riders on public transit, referring to the “drunk Indian” stereotype (43F, Vancouver); to being discriminated against by members of their community and removed from stores, restaurants, coffee shops, and other public spaces, sometimes by threat of calling the police:

*So, yeah. It’s keeping me…It’s been an impact where it’s harder to do things […] and I been being discriminated everywhere I go. I try to go to like certain rest…stores, even just using this simple bathroom, some places they just turn me away. I been turned away from coffee shops. I would like, go in there to get a coffee, and they start bugging me and things like that. And they keep—they’ve actually told me, when I wasn’t even doing anything, they told me, ‘Oh you gotta leave,’ and I’m like, ‘What? I was just trying to buy a coffee!’ and then they’re like, ‘You gotta leave or we’ll call…’ and I’m like ‘Oh my god.’* – 32M, Vancouver

Participants felt that violence was already a regular occurrence in both study sites. However, higher stress and uncertainty, as the toxic drug crisis worsened and pandemic restrictions forced people to use alone, caused greater unrest for everyone. For those living in the downtown areas of both cities, substance use had been wrought with life-threatening uncertainty for over a decade, and the circumstances worsened during the pandemic, causing “people [to be] out of control and starting to not care about themselves.” (27F, Prince George). COVID-19 protocols restricting access to safe spaces and services forced Indigenous women into unsafe situations and put them at greater risk of experiencing violence. One woman described the dangers of being killed or going missing for all Indigenous People, but particularly women and girls in Prince George, personally knowing several women to be found murdered or disappeared completely since the 1990s. She said that since the pandemic started, means of safe travel put in place to try to protect Indigenous People from abduction were curtailed and some of her friends and family resorted to hitchhiking along the Highway of Tears (724 km length of Yellowhead Highway 16 in British Columbia where many Indigenous women have disappeared or been found murdered) between Prince George and Prince Rupert, and have not been seen again.

### Police interactions

When asked about interactions with the Vancouver Police Department (VPD) during the pandemic, Vancouver participants brushed off instances of “routine” street-checking for being out late at night during COVID-19 restrictions. In a city where the police force is known for harassing and arresting disproportionately higher rates of Indigenous Peoples, particularly women [41], this ongoing, normalized street-check behaviour is concerning. Other similar experiences included mistaken identity; instances where police racially profile young Indigenous men and arrest them using excessive force:

*Fuckin’ one time, I dunno if they made a mistake, but they fuckin’ all surrounded me and then they pulled guns on me, I thought I was—I was wondering what’s going on here, and then they looked at me and then they’re all like ‘It’s the wrong guy,’ and they got up and drove away, and I was like ‘Hol-ay fuck.’ […] But they were looking for somebody eh? And they thought…I must have looked like the guy they were looking for. And then fuckin’ they had me down, and I’m fuckin’ layin’ there looking around like, fuck. And then they looked at my face and ‘It’s not the guy eh?’ they all packed up and left and I was still laying there like, whoa.* – 32M, Vancouver

When asked about interactions with the Royal Canadian Mounted Police (RCMP) in Prince George during COVID-19, multiple participants spoke of violence and shared feeling helpless in the face of police brutality because, even before the pandemic, “they get away with a lot of shit man, they get away with murder and everything, it’s hard to stop them right.” (30M, Prince George). During COVID-19, participants shared stories of police engaged in racial profiling and racist behaviour and treating unhoused IPWUD with callousness and aggression sometimes leading to severe injury or death. Troubling experiences included losing friends and family to police violence. One woman spoke of how the RCMP tried to minimize or redirect responsibility for the death of Indigenous People in custody: “One of my cousins in [home community] died in city cells, and they say that he died of withdrawals. That’s bullshit.” (42F, Prince George). Another participant in Prince George shared that the police allegedly killed his friends during the pandemic: “They make dogs chew on them and shit, they kill them in the cell, and pepper spray them ‘til they die and all that kind of shit. They just kill them. […] beat them to death and shit. It’s all fucked up.” (29M, Prince George). His friend allegedly was attacked by a police dog on the street and died from infected wounds a week later. Police were also known to cause harm and even death when conducting routine checks or responding to nuisance calls, according to participants. Another participant in Prince George shared that she was bullied by RCMP officers who responded to a call during stay-at-home restrictions about a conflict between her and a neighbour who had been harassing her. She had repeatedly asked housing staff to help her de-escalate the situation before it amounted to violence, but they were not proactive about the situation, and police were called when the conflict became aggressive. Officers on the scene showed no effort to resolve or de-escalate the conflict without resorting to physical measures:

*When the cops come around the corner, and they attempted to arrest me, I was [saying], don’t touch me, don’t touch me. And I kept pulling my arms away from the female officer. The female officer could not detain me. She called for backup. When she called for backup, that was when I had a male, skinny little male white officer come and put his fucking knee [in] my back, which I didn’t appreciate, then put to his, then put the handcuffs on me. And then they walked me up to the fucking cop car in my underwear with my pants down to my fucking ankles. So it embarrassed the fuck right out of me.* – 39F, Prince George

The police officers resorted to physical restraint, “jerseying” her by pulling her shirt over her head and not helping her when she asked them to fix her pants that were falling. Other participants in Prince George shared stories about police officers destroying their private property during COVID-19, such as tents and smoke pipes. One participant said that a police officer stole his welfare money; another, her necklace and engagement ring given to her by her fiancé. Both said that the officers involved assumed they had stolen those things and offered no recourse for retrieving their belongings.

Participants felt, particularly at the start of the COVID-19 outbreak in British Columbia, that IPWUD were at greater risk for COVID-19. Many COVID-19 symptoms overlap with dope sickness—symptoms of withdrawal from opiates—and introduced the possibility for someone to unknowingly transmit a dangerous respiratory virus to vulnerable friends and family members. For some, this realization did not come until much later in the pandemic: “I do a lot of heroin on the street, at the time I was on some, I got COVID, and I must’ve thought I was dope sick and shit. Yeah. And that’s probably why I didn’t know I had it, right? So. I didn’t even realize I was sick I was just so dope sick most of the time.” (38M, Prince George). Another participant shared that she was sick but never knew the exact cause: “I was sick, but I believe that could have been, like, because I’m a drug user. It could have been drug use. Could have been alcohol. It could be the season. I don’t know.” (34F, Vancouver).

### Cycles of isolation and crisis

Participants who were in crisis frequently had no place to go: to eat, sleep, shower, work, for ceremony, or to live safely. When in-person services shut down at the onset of the COVID-19 pandemic, there were immediate and serious impacts on people who relied on them, particularly when it came to food and shelter. Participants in Prince George found it particularly difficult to find a place to eat during the winter months, when gathering and restaurant restrictions prohibited drop-in centres from allowing people to come inside to eat their food. Participants were forced to take their food outside and find locations to eat where they were not at risk for harassment or aggression from store owners:

*Well, [the drop-in centre], they’re just open for food, so you just go there and get your food, and then you have to leave, so that would make it hard for us to eat. Outside. Cuz you know, businesses don’t want you eating out there, outside their business, right, so it’s… tryin’ to find a place to eat is hard.* – 38M, Prince George

### Connection is survival

Participants felt that the COVID-19 restrictions exacerbated pre-existing vulnerabilities by cutting off vital connections that supported their basic needs, such as access to food, shelter, and social support networks. Forced isolation put IPWUD at greater risk of overdose and other health crises like self-harm and suicide. More than 40% of the participants in this study have overdosed at least once since the pandemic started, and most of them have overdosed more than once. Being “narcanned” with Naloxone, the emergency antidote for an opioid overdose, has become normalized in everyday life for Cedar participants, who are regularly witnesses to or responding to overdoses in addition to being at risk themselves. The toxic drug supply has introduced regular uncertainty and stress for people who use substances, especially those who are forced to use alone:

*Just you don’t know what you’re getting [in the drugs]. And there’s times where, like, so many times where I could have died. And like there was nobody out and about or around or anything like that. So I mean…So the fact that I’m still alive is pretty cool. Like, I mean, there’s gotta be something out there. That’s looking out for me. But yeah, so I was using the fentanyl and whatever else was mixed in with that is what I’m saying.* – 42F, Prince George

Those who have survived overdoses shared this sentiment of good luck mixed with timing, where they are more likely to survive simply by using in the company of others in case they administer a toxic dose. These experiences underscore how connection is not only essential for meeting daily survival needs but also plays a protective role against harms like overdose. One participant shared a kind of spiritual experience while overdosing, that he survived because his brother just so happened to show up the moment he needed help: “he said I was sleeping on the couch but he couldn’t tell whether or not I was breathing or not but my eyes were still open.” (28M, Vancouver). Participants relied on their families or romantic partners for help during overdoses: “She [girlfriend] would do it she’s brought me back a few times, but just by breathing, um, a friend of mine narcanned me eleven times! Just, right beside us, at the legion.” (38M, Prince George).

For those who did not live with significant others during lockdown restrictions, the consequences of an overdose were more severe, forcing some people to try to reduce the regularity or dose, or quit using altogether: “Oh, yeah, it’s in the past. One of the reasons I’m getting clean now. Yeah, I don’t want to die.” (43F, Prince George). Participants found that substance use increased at the start of the pandemic, but many have cut back as they witnessed their loved ones passing away from overdose because of being in isolation: “some family and friends that passed on. So you know that, that got to a lot of us [who used alone more] because of COVID or being isolated, you know, quarantined or whatnot.” (34F, Vancouver).

“I’m pretty sure that it’s definitely gone up because people are using by themselves.” (38F, Prince George). Most participants blamed quarantine and restrictions for the spike in overdoses since COVID started, which contradicted some protocols for the concurrent toxic drug supply crisis that included never using alone. Restrictions on social gathering forced people to stay home alone, and most participants shared that people they know including friends and family not only were forced to use alone, but were more likely to use more as a means to cope with loneliness and the stress of isolation: “Yeah I think it is higher ‘cause a lot of people are staying home. ‘Cause they don’t wanna go out and then they’re doing drugs and they can’t handle it, just fuckin’ die. Lot of people have been dying eh.” (32M, Vancouver). Participants also felt that their own risk of dying from an overdose increased with quarantine and lockdown restrictions. Isolation was not the only driver of the increase in overdoses; participants shared that the supply of drugs has changed over time to the point where they can never be sure of the contents of what they are taking:

*The coke you get nowadays is not even worth it. It’s like, 80% cut, 20% coke right? Back in the day, I would do a 10-point of coke and a 10-point or half-point of down and I would OD. ‘Cause it was strong, right? Now, fuck, you get that shit today it’s not even, you don’t even get a buzz.* – 43F, Vancouver

### Community and culture

Travel restrictions impacted participants in this study. Most participants in the Cedar Project are from Nations far away from Prince George or Vancouver, but some have lived in these cities for a long time or their entire lives: “I’m a concrete Indian. Born and raised here in the city.” (49M, Vancouver). Many Cedar participants living in the downtown areas of Vancouver and Prince George rely on being able to go home to their community for special occasions, ceremony, to harvest, and visit family. When travel restrictions impacted personal and public transit to regions outside the downtown areas of both study sites, many Indigenous Peoples were cut off from their communities unless they lived there full-time. For those who had to travel by air or water, it was especially difficult. One participant shared about not being able to return to Vancouver Island for ceremony:

*It’s hard because, like I said, we can’t do our potlatches, we can’t do a lot of stuff that we used to do, like our soccer tournaments in [her community], we can’t do stuff we were normally able to do ‘cause of COVID. So it’s kind of annoying. And I’m a traditional person I like going to those kinds of ceremonies.* – 43F, Vancouver

Closures of important community spaces and transportation options kept participants in this study from accessing vital resources like places for ceremony, personal hygiene, and safe spaces. Many were left stranded without access to services they depended on when the pandemic started: “uh, it was horrible. We couldn’t use the washroom anywhere. We’re barely, it was basically like we weren’t allowed to go in anywhere.” (27F, Prince George). Places to keep warm and stay safe became unavailable to folks in both cities, but Cedar participants in Prince George faced additional challenges due to their harsher winters. For those who use substances in Prince George, access to the needle exchange (a central establishment in downtown Prince George, important to the lives of Cedar Project participants) was restricted: “We’re not allowed in anymore; they give us like, supplies and stuff, but we should be able to sit in there all day.” (42F, Prince George). Similarly, the Drinkers Lounge (a community-managed safer consumption of alcohol program in Vancouver, BC), had to limit their regular, well-attended cultural activities: “We just have drummers coming in. We have Elders, it…it’s been really messed up since this COVID. You know, especially for them dealing with their family and friends on the outside, and their office and other areas to go drumming.” (34F, Vancouver). Not only were gatherings and performances restricted due to COVID-19 transmission risks, but drummers and Elders had unique challenges to deal with during the pandemic as well.

### Housing first

Participants in this study felt that housing efforts failed them during the pandemic. In Prince George in 2021, the city passed the Safe Streets Bylaw [42], a controversial piece of legislature that allowed bylaw officers to ticket people for “nuisance” behaviours like setting up shelters, laying down to rest, loitering, panhandling, and openly using drugs on public streets.

*I mean, as for actually getting people off the street and stuff like that, it didn’t really go too well. […] With everything being closed down, people were […] looking out for themselves. But you know, we sort of became the things that didn’t matter. Like people didn’t really care.* – 42F, Prince George

The strain felt by unhoused participants impacted all aspects of their life: “I don’t know if it’s just stress, I just wanna, like I need a place to stay, and just not having a place to stay it sucks.” (32M, Vancouver). Participants without stable housing had a hard time finding regular rest; between having to find safe places to sleep, protect their belongings from theft, and perpetual harassment from city bylaw officials, many are critically exhausted. During several of my interviews, participants gradually fell asleep once they had eaten, had a coffee or hot chocolate, and relaxed.

*I just stay here and there, that’s why I got a bike, so I can some nights I just ride all night eh? I don’t wanna lay down, but I do get tired, so I just pull over in certain spots in nighttime where there’s hardly nobody. And I get off my bike, and I lean against something like this [leans back] and put my legs on my bike so that way anybody tries to take it I’ll know. I just get a couple of hours rest, more—more meditate more like, that’s all I can do I don’t wanna fall asleep sometimes. There’s guys just watching to see if you fall asleep like and take your shit.* – 32M, Vancouver

The perpetual cycle of crisis and instability, and the need for housing before all else, was epitomized by the quarantine housing protocol for the unhoused population when they were sick with COVID-19 in Prince George. Several participants shared that during COVID-19 they spent time in quarantine, housed in modified hotels where they were able to stay warm, eat, and in some cases reduce their substance use before being returned to living outside once their 14-day quarantine period ended. One participant called out this problematic model that contradicts the political rhetoric about providing stable housing and recovery support for people like her:

*I got put in this one over here [shelter]. But then right away, I was, and then those were times I was able to get a little bit clean and get started. And then right away, I went from there right back onto the street. So I was back out using within the day. Which is, which is fucked up because you know, they [say], okay, well, let’s help them with housing, you know, give them some methadone and get them going. And then they’re trying to say that they’re helping us, and then they just put us out on the street right after we’re done.* – 43F, Prince George

Participants who were unhoused were left without places to clean up and follow enhanced hygiene protocols, which impacted their perceived risk of contracting COVID-19 while having a cascade of other impacts on their wellbeing: “You know, like, plus even getting into a grocery place with looking the way that we do because we can’t wash up good enough when we’re out there.” (42F, Prince George). In Prince George, several participants felt jarred by the immediate lack of support when the pandemic began:

*It was pretty rough. I mean, things just totally shut down. And when it first started, I was still in my addiction. So I mean, it became, like, doubly difficult to live life. Like, I know I lost a bunch of weight. Like, way more so than I would have without, like. It was hard to find anywhere to go to the bathroom to get cleaned up. I know that my personal hygiene went down the toilet because I was living on the street. And there was nowhere, everything was closed. So I mean, it was horrible. They said they were gonna give places for people to live. And that never seemed to happen.* – 36F, Prince George

Another participant who was unhoused at the time of the interview shared: “I do that [wash hands] a lot anyways eh I am a pretty clean person I always wash my hands and my face. Like to have a shower. Keep clean.” (32M, Vancouver). When asked where he goes to access these resources, he said: “just anywhere, right now I need a shower today, so I might just go over to [local shelter] here and sign my name and go have a shower there.” The personal impacts of the pandemic were highly dependent on participants’ level of comfort in unstable, transient circumstances and their knowledge of where to find resources. In Prince George, where fewer services are available than in the much larger city of Vancouver, participants more often shared challenges in having their basic daily hygiene needs met. Overall, participants shared that they easily changed their hygiene behaviours to include more hand-sanitizing as it was widely available in public spaces, considering the risk of catching and transmitting COVID-19.

In addition to housing, having access to a regular means of communication was critical to feeling stable, staying in-the-know about changing circumstances and restrictions, and connecting with friends, family, and loved ones. For many participants, family lived too far away to visit regularly or to be able to visit under the COVID-19 travel bans, so online messaging, social media, and videoconferencing were vital to staying connected—all of which require access to a phone or computer. Yet, many participants were consistently without a phone throughout the pandemic. For a participant from Prince George who was recently housed at the time of the interview after years without stable housing:

*I would just say like, if I was back out there on the street, […] not having a place to live. And like people who are, who are homeless and that are way more at risk than anything. Um, and not just that, but I mean, being in high-risk situations and not having the means to have their own phone, or, you know, like, even if they had a place to live. I mean, you have to be able to have a phone or some sort of Wi-Fi or something so you can keep up on the news and stuff like that, right?* – 43F, Prince George

### Fear and trauma-driven distrust amplified experiences of grief and loss amidst conflicting public health protocols

Emotional responses to COVID-19 are rooted in intergenerational trauma and colonialism, causing severe grief from pandemic-related harm and the ongoing toxic drug crisis. Experiences of grief and loss predated the pandemic for Indigenous Peoples in British Columbia due to concurrent and ongoing health emergencies, and these emotions became more intense when layered with distrust in the colonial response to COVID-19. Some participants believed sociopolitical circumstances and the spread of infection could get a lot worse because federal and provincial governments continue to shirk responsibility for injustices and upholding our rights as recognized by the BC Declaration on the Rights of Indigenous Peoples Act [43]. Some felt that the pandemic could have been avoided or the effects mitigated had the federal government acted sooner to prevent the spread of COVID-19.

### Safety

Some participants were scared for their personal safety, the safety of their loved ones, and their broader communities and believed COVID-19 would be the harbinger of social collapse. A woman in Vancouver shared her deep fears when the pandemic was declared:

*I thought we were all gonna die…Like, me and [her partner]. And everybody else that was in the Downtown Eastside. I thought everyone was gonna die there. I thought we were the first people that were gonna die.* – 26F, Vancouver

She was deeply fearful for herself and her partner, and her children who lived with her mom. She felt that COVID-19 was going to be a “very deadly kind of virus that no one can get rid of,” triggering a mass extinction event. Others shared a similar sentiment that COVID-19 was never going to go away. For PLHIV in Prince George and Vancouver, they feared they might not survive if they got COVID-19 and were careful to follow all restrictions and stay home as much as possible. Similarly, participants with family who have chronic illnesses, particularly chronic obstructive pulmonary disease (COPD), were careful not to risk their loved ones’ safety.

Few participants had strong feelings about having to wear masks; a few called masks a difficult adjustment that they were willing to make but wished others around them were more willing to follow mask protocols. A woman in Vancouver living with HIV shared frustrations about her fellow public transit riders not following mask mandates or extending their use once mask requirements were lowered by the province. She alluded to the rising anti-mask movement and Freedom Convoy mentioned previously when she said, “’Cause like, they could be…you don’t know if they’re just not wearing a mask because they’re not vaccinated or like, because …” (43F, Vancouver) highlighting a key turning point in 2022 when not wearing a mask came to mean someone might be a supporter of the growing anti-pandemic restrictions movement [44].

### Distrust in colonial authority

The pandemic exposed a deeply rooted distrust of colonial authorities and information sources, rooted in historical and ongoing colonial practices that have consistently ignored Indigenous Peoples’ rights and autonomy. Participants who lacked trust or feared colonial authorities were more likely to look to their local networks and communities for information, which sometimes led to the circulation of false or outdated information and perpetuated skepticism towards colonial public health measures. For example, a participant in Prince George was unsure of where to look for information:

*Um, you’d hear all sorts of different things. […] Like, just like with anything, right? And then it was changing so much, and it was working so fast that you didn’t really have time to figure out what what was really real and what wasn’t, right? And I mean, except for the having to wear a mask. And just how quick it went around the world. Like, I mean, it was pretty, pretty phenomenal, how fast it went across the world. This is why I think that it was, didn’t just start in one place. I don’t think. […] You don’t really know, um, who would, like, who’s telling the truth and who’s not, right? Because I mean, like, things in the media are so, there’s so many people who don’t tell the truth. Like you just don’t know anymore.* – 36F, Prince George

Common conspiracy theories included that COVID-19 was a government hoax to cover up something larger, perpetrated by any number of nations but most commonly the US government. Some felt it was a distraction from aliens, and that lockdown had a different purpose entirely. Others felt that it was a mechanism of population control; that the death toll was real but deliberate, or that by some other biological mechanism government authorities were trying to supplant control over peoples’ bodies, or turn us into “some weird looking creature,” like a zombie (28M, Vancouver).

*More things are gonna happen. They got something planned, something we don’t know about yet is gonna happen…Whoever’s controlling things, you know like there’s always people controlling things out here. Yeah…I think it’s gonna get worse. There’s things that people know like, all the countries the whole world knows about but, they don’t tell us. They don’t tell us right. They don’t tell us the truth.* – 30M, Vancouver

Many simply felt that the pandemic was blown out of proportion and that there was fear mongering involved, like a political tactic at the expense of constituents’ peace of mind.

Experiences of abuse throughout life made it difficult for people to trust others, whether in personal relationships or people in positions of authority, which created challenges for communicating critical public health information. One participant shared:

*There’s lots of places where I’ve been treated so badly, and that I’d have to say from, like, people that I’ve known quite a while that I figure wouldn’t lie to me. […] But I go with the people that I’ve known the longest, you know? Like, when I hear from pretty reliable sources, that wouldn’t usually steer me wrong, which are people that I’ve known for a long time. So yeah.* – 43F, Prince George

She was protective of herself and her network because of experiences of abuse and breach of trust. Other participants shared a similar lack of trust in the people around them. Even trust in people infected with COVID-19 was called into question: “I guess, well, some people say they have [contracted COVID-19]. I don’t know if they really have.” (29M, Vancouver). This was particularly frustrating for those who have lost loved ones to COVID-19; one participant called out confirmation bias among “non-believers” who never knew someone to get seriously ill or die from COVID-19:

*Well, like there were some people on one side and some people on the other [believing COVID-19 is real or not]. A lot of the people that were on the side of believing, though, has lost people from it. And like, so I mean, the idiots that weren’t directly affected, are constantly thinking… [it’s not that bad]. And I mean, to be honest with you, at first, I thought it was bullshit too. Until, like, you know, until I realized like, you know, people are actually like, were actually dying, you know?* – 38M, Prince George

Others echoed that skepticism until they were proven otherwise:

*Everybody’s like this is a hoax or whatever. Next you know, like everything, the whole entire fucking, everything was completely dead silent. I was like, I was like what the fuck. I was like looking around, there’s like nobody on the streets. This is fucking weird. I was like, I gotta get, I was like, I can’t be around this area. (Laughs) Because that’s when I was down on Granville. I was walking around, it was so dead. […] It was, it just felt like it, like as if, like, um, you know, how they have those scenarios in movies where you go to sleep in the hospital, you wake up and then all sudden the whole entire world gone. It’s just you and like a couple other people. Yeah, that’s what it felt like. It was fucking weird man.* – 34F, Vancouver

### Grief

Through frequent loss of loved ones, and threats to daily life through constant overdose risk and risk of infection, experiences of trauma and grief were severe. All participants shared grief and sadness over lost friends, family members, and intimate partners during COVID-19, due to infection—particularly prior to vaccine rollouts—or to the toxic drug crisis. Lives were frequently uprooted by death and mourning: “I’m feeling just isolated these days. […] And I keep doing what I’m doing, what I used to do. I guess I just try and keep a smile on my face.” (28M, Vancouver). One participant shared that she was “losing a lot of family and friends through COVID and non COVID” and struggling with “not being able to be face to face, like contact with anybody that we’re usually hanging around with day to day” (34F, Vancouver).

One participant in Vancouver used to meet his social needs among work friends but given the precarity of the type of work they did as carpenters and journeymen looking for cash jobs, and that most of them were unstably housed, the impacts of the pandemic were extreme: “I used to go to work…every morning. Meet up with a couple other buddies, just cash, cash corner jobs. Now it’s empty. They all passed away.” (28M, Vancouver). “Cash corner” refers to an area in Vancouver’s DTES where off the books, “labour ready,” workers hang out and wait for odd jobs that contractors need done, typically for cash pay. Several participants lost their best friends during the pandemic and found it extremely difficult to manage their grief in isolation, having detrimental impacts on their mental and spiritual wellbeing: “I think I broke my mind” (29M, Vancouver), shared one participant after telling me he watched his friend die from an overdose, unable to help him. Another lost his daughter to an overdose during COVID-19, and dealt with feelings of guilt, anger, and self-blame for not being able to help her. Another participant in Vancouver described the overwhelming rate of loss, crippling her and other survivors’ ability to mourn properly: “it’s like we lose three at a time during one week or else four just during Mother’s Day weekend was four in one week. And to me that was like overwhelming.” (34F, Vancouver).

Another lost two sisters, two friends, and a brother-in-law since the pandemic started—all but one to the toxic drug crisis. One participant talked about being incarcerated when the pandemic was declared and being released to empty streets with nowhere to go. Another witnessed a stranger die by suicide while staying in a quarantine hotel on Vancouver Island. Others were already dealing with intense trauma and grief when COVID-19 hit; one participant in Prince George lost his father to cancer, and the death broke the family apart and he began using *down* (fentanyl-based street drug, usually with an unknown composition of illicit and toxic drugs and filler agents) to cope. He became homeless shortly after and lost contact with his brothers and community.

Participants with romantic partners dealt with a variety of hardships during the pandemic, and often had to help their partners in mourning while managing their own grief. A participant shared her concerns about her partner’s mental health after losing his best friend to overdose during the pandemic. She worried that he was shutting down and using more, making it difficult to communicate and help him. Some participants shared histories of self-harm or attempted suicide before the pandemic, amplifying the intensity of the strain the pandemic placed on people already in crisis. One man lost his fiancée to COVID-19 before vaccines became available and has nearly died from overdose three times since.

### Resilience and connection were important buffers against pandemic-related harm

Being connected to those around us was protection against isolation during COVID-19, helped mental health, and kept people safe. Connection was an important buffer against the stresses of precarity and isolation during the pandemic. For one participant, the onset of the pandemic was a positive time in his life:

*…It was going good for me, for starters it’s the first time I’ve had my mum back in 20 years, so I stayed with my mom at [lodging], so that’s the first time I’ve met my mom in 20 years, so I was happy to see her and stay with her…Starting to know my mom again, so I’m just happy.* – 38M, Prince George

### Mental wellbeing

Reconnecting with family was a morale boost for him at a time when grief from isolation was reaching critical levels. When able, some participants leaned on their parents, brothers, and sisters for support if they lived nearby. For others, the pandemic triggered traumatic memories and conflicting emotions about reconnecting with family who had previously been estranged, out of fear of imminent danger for themselves and their loved ones:

*I got so scared, and I was like, flipping through like…sending my mom messages and stuff, telling her that I loved her. Sending my mom—I didn’t have my brother on my Facebook—well I did, but he was not messaging me at all yet. Like, he didn’t want to talk to me at all yet. He was holding a big grudge on me because of my lifestyle and my life choice. But I’m slowly getting out of it now. Um, COVID had a big impact on me. It scared me a lot.* – 26F, Vancouver

COVID-19 restrictions limited access to community and social interactions with friends and family, particularly for kids and older adults who were used to regular social activity during the day. For one woman living with her child and her mother, she shared concerns about their coping with isolation and negative impacts on their mental health and wellbeing: “When I went back home, it was a big change for them, like my boy’s not going to school anymore, and he stays home all the time and just smokes weed and plays games, and my mom stays in her room drinkin’ all day.” (42F, Prince George). Others were similarly worried for their own wellbeing and ability to handle restrictions on socializing:

*It’s been okay, some of it’s been a little stressful, um, due to the fact that not being able to really be in contact with some of my friends, but I have just made my circle a little smaller, and remain close to them. Not being able to visit with my family on a regular basis […] I’ve also been bullied a little bit in the place of residence that I’m living at. So it’s kind of been a little bit of a hard last year and a half for me, not the first little while, but the last year and a half has been really rough for me.* – 39F, Prince George

Many Indigenous People have been unable to attend regular cultural groups, women’s groups, or attend important milestone events and ceremonies with their families and communities. For example, one participant shared having to miss all her children’s birthdays since the pandemic began:

*Not being able to have actual social events and gatherings and like birthday get like, say birthday celebrations, because there’s been a couple of birthdays that’s going on. Like, actually, for the last two years, I’ve missed all my kids’ birthdays because I can’t celebrate them with them.* – 40F, Prince George

### Safety net

Participants shared a general sense of needing to look after themselves and not being able to trust or rely on others, especially since the COVID-19 pandemic started: “I just feel that I have learned throughout this pandemic that the only person that’s really truly going to be there for yourself is yourself.” (39F, Prince George). Heightened safety risks during COVID-19 exposed critical gaps in social safety nets among loose acquaintances and social networks built upon substance use, trauma, or survival-based activities. One of the main reasons shared for not feeling like they could rely on their friends for help was that their friendships centred on substance use, and that was everyone’s top priority.

Participants with more positive perceptions about their social network and personal relationships were more likely to feel like they would be supported if they needed help. One man in Vancouver shared that his girlfriend is a “positive person” for him, that she “encourages [him] to do good.” (29M, Vancouver). Women frequently shared the importance of ongoing weekly social and cultural groups, worried about when or if they would be closed. Some group activities closed permanently, while others transitioned to online platforms, but most hoped to return to in-person gatherings when it would be safe. Social support networks included neighbours they could turn to when they were having a hard time during the height of COVID-19 lockdowns: “When I start getting suicidal, I go downstairs, to where [they] live and […] I just sit around with them and bullshit with them until I calm down.” (36F, Prince George). One participant shared that she regularly helped her upstairs neighbour who had chronic health issues with dishes and other chores. Others, particularly in Vancouver, shared that their friend groups collectively watch each others’ belongings when they were away from home or their camp.

### Coping strategies

Participants with pre-established routines and stability that did not depend on socializing or activities in public spaces were better equipped to weather the restrictions at the onset of the pandemic. Others “had to pretty much drop everything when COVID hit,” (36F, Prince George) and some turned to substance use to cope with idle time, loneliness, and uncertainty during the pandemic. A woman living in the DTES of Vancouver shared: “I drink. I do drugs. But it’s like daily, I really like seeing, being around my family and friends.” (34F, Vancouver). She said that she leaned harder on substances when she was unable to be with her friends and family. Continuity of access to routine care like safe supply and moving to virtual forms of connection when the pandemic began helped mitigate certain risk factors for overdose during a time when people were more likely to be using; despite this, having to use alone remained a salient risk factor.

People engaged in regular in-person gatherings for ceremony, cultural activities, and mental health support were negatively impacted by pandemic restrictions. Many participants in this study relied on group activities in public spaces for their wellbeing and were negatively impacted by lockdown measures:

*I was in an art class. Aqua fit. Volleyball. Um, and there’s a couple more. I had a job as well. There’s one other thing I can’t think of. I had to drop everything because of COVID. So I’ve been stuck at home doing nothing. It’s so boring. I gained a lot of weight. My mental health is horrible.* – 36F, Prince George

For others, mandates to stay home and not attend social gatherings were more aligned with their typical behaviour before the pandemic: “I’m okay with not going out. I’m okay with not going out to dinners or having a social life like that. I’m okay being home every night by myself.” (43F, Vancouver). Some folks preferred to be on their own and felt more fear for others’ safety and well-being rather than themselves. People who perceived themselves to be naturally introverted and stable in their personal routine were less impacted by disruptions to social life during the pandemic. Intentionally maintaining a positive mindset and a positive sense of self was a helpful coping strategy during COVID-19:

*My coping strategies are just to not look at it look, not look at things negatively, try to look at the positive outlook on things. And that’s what has gotten me through a lot of my struggles in life is just looking at the positive in things. Because I have, I’ve had a lot of barriers in my life. And I mean, a lot. So it’s not just the pandemic, it’s been throughout my whole entire life. And I’m 39 years old now. So, I’m a survivor. I’m a warrior. And I’m a fighter. It’s made me stronger, and it’s made me who I am today.* – 39F, Prince George

Others chose to pay special attention to their physical health to stay well during the pandemic. Going for walks, watching movies, and relaxing by themselves or with intimate partners, and staying on top of exercise routines and healthy eating helped some Cedar participants cope with restrictions imposed during the pandemic. Some participants were able to stay engaged with harm reduction or recovery programs.

Arts-based and culture-based resources for coping with loneliness and stress helped participants maintain a positive sense of self. Some cultural activities were curtailed during the pandemic, like drum circles and in-person Elder support, while others were more amenable to self-isolation, like smudging and arts-based activities. Some participants found comfort in artistic pursuits they already had before the pandemic, and others felt exhausted by them: “I used to colour a lot, but I still collect the colouring books. I just haven’t had the time or the oomph to do it.” (36F, Prince George). Other activities Cedar participants engaged in throughout the pandemic included carving, beading, drawing, graffiti, making jewelry, cooking, and learning new skills online through YouTube videos like aesthetics and makeup. All these activities were sources of positivity and strength for people struggling with isolation during COVID-19, and for some, a source of additional income to help with rising costs of living and scarcity of employment options. Only one participant, in Prince George, shared that she leaned on her religion for support during COVID-19. Multiple others shared feeling despondent about the state of the world and losing faith in their cultural or spiritual beliefs as a result. These participants reported worse mental health during COVID-19, some shutting down completely and leaning on substance use during lockdown.

## Discussion

Through this exploratory qualitative study, we aimed to gain a deeper understanding of the experiences and survivance of IPWUD during the COVID-19 pandemic, in the context of concurrent and conflicting public health emergencies including the toxic drug crisis, housing crisis, and increasing number of environmental and economic emergencies [45,46]. COVID-19 and the pandemic response increased stress and fear for safety and future survival. The pandemic tested survival skills and Indigenous Peoples’ ability to protect themselves and loved ones, because it has made daily life harder and less predictable. New evidence continues to emerge that suggests reinfection with SARS-CoV-2 significantly increases risk of hospitalization, long-term health impacts including pulmonary, cardiovascular, and gastrointestinal disorders, and death [47,48]. Yet, when asked about the hardest parts about the COVID-19 pandemic, most participants did not mention concerns about the impacts of infection at all; rather, precarious circumstances and increased instability were the most common sources of stress and difficulty during the pandemic. Additionally, the In Plain Sight Report—whose release coincided with the onset of the pandemic—found alarming levels of anti-Indigenous racism across healthcare institutions in the province [49], highlighting an additional risk for Indigenous patients requiring hospitalization for COVID-19.

During times of crisis, it is common for people to act out of fear and look for others to blame for their current circumstances [50,51]. When the pandemic first started, there were pockets of disbelief or doubt in the severity of the spread of infection, and skepticism about the circumstances surrounding COVID-19 among downtown communities in Prince George and Vancouver. Deciding COVID-19 was a hoax because one grew tired of public health restrictions is different than genuinely believing something larger or malicious is at play. Skepticism and distrust in colonial authorities are not without cause. Intergenerational and complex trauma are the result of historic and ongoing colonial oppression and violence in healthcare. Indigenous Peoples have endured over a century of residential schools, day schools, and Indian hospitals, often being the subjects of experimental procedures and medicines, including vaccines [11,52]. Today, many Indigenous Peoples experience intergenerational trauma, different forms of abuse, broken families, substance use, the direct and indirect impacts of historic and ongoing colonial policies of oppression, control, and genocide and are significantly more likely to be forced into the foster care system [6,11,53]. Thus, decision-making in the context of the COVID-19 pandemic among Indigenous Peoples in the Cedar Project is rooted in trauma, grief, and intergenerational survival mechanisms.

Research and historical records have shown that discrimination spikes in the aftermath of disasters or major emergencies, and the COVID-19 pandemic was no exception [51,54,55]. Police brutality is a major ongoing concern for Indigenous Peoples, as the original enforcers of the Indian Act and perpetrators of racism and violence since long before the COVID-19 pandemic started [56,57]. Police violence is an ongoing issue particularly for Indigenous Peoples living in Prince George [58], where Indigenous People represent more than 80% of the unhoused population [59]. With the implementation of street-sweeping bylaws [42], the increased presence of RCMP is extremely triggering for Indigenous Peoples with intergenerational and complex trauma and PTSD.

Participants who expressed confidence in their personal coping strategies (e.g., “I just keep doing my own thing”) seemed more resilient in the face of pandemic-related hardships, possibly because they were able to protect themselves during crisis by more readily adapting to changing circumstances. This has been documented in Indigenous populations elsewhere, where despite historic and ongoing sources of trauma, Indigenous Peoples persevere and live relatively fulfilling lives, helped by a positive sense of self or self-confidence, strengthened by cultural identity [60,61]. This includes participants for whom their routine was not as disrupted by pandemic restrictions as others, but more importantly it includes people who have found *any* way to cope with challenges they faced and survive periods of crisis. Those who relied more on external and social resources for mental, physical, and spiritual wellbeing seemed to cope less well (e.g., report worse mental health) than those who were more accustomed to keeping to themselves. Resilience should not be considered a moral triumph or failing, however. In part, resilience refers to both adaptations and steadfastness in the face of adversity [13,62]. For participants in this study, sources of resilience were highly individual. Ultimately, participants’ resilience during COVID-19 contributed to life stories of survivance, which might share similarities only in the ancestral knowledge that is woven throughout all of us, shaping our lives and the lives of IPWUD in the Cedar Project during COVID-19, through the myriad ways we all coped with the challenges it brought.

These findings exemplify Indigenous survivance, as the culmination of historic and cultural factors and the depths of intergenerational knowledge that are drawn upon to support wellbeing during health emergencies. This can be seen in Cedar participants’ priority setting, internal and external resourcefulness, and most importantly, recognizing that during an emergency, any means of coping–and surviving, resisting–is better than none. Using a survivance lens exemplifies strengths-based research; Cedar participants experienced tremendous hardship yet maintained hopefulness, and love for themselves and their families. Taking a strengths-based approach to research does not mean that negative experiences are filtered through a lens of positivity, but rather that the message of survivance during these experiences does not go unrecognized. Survivance can be seen in how they have maintained who they are and who they want to be amidst the challenges presented during the pandemic.

Participants’ fears expressed in this study are a collective mirroring of what is expected of them as IPWUD, a minoritized and problematized population from the white settler gaze, where complex health challenges due to colonial oppression are conflated with inherent characteristics of being Indigenous [63]. Internalized—and externally reinforced—deficits-centric beliefs about Indigenous Peoples’ vulnerability to disease and overall poor health may have contributed to the expectation among participants that IPWUD would get very sick or die from COVID-19. Throughout the pandemic, and specifically in the DTES, IPWUD did not “die off from COVID” like one participant feared; rather, the community persevered and demonstrated their ability to look after each other and do what was needed to stay alive. To understand this as survivance, is to understand and respect Indigenous knowledge as valid ancestral (e.g., time-tested and intergenerational) and collective (e.g., inherently community-focused) knowledge that would greatly benefit health policy and decision making in an emergency not just for Indigenous populations, but for all people. What Indigenous Peoples and communities need most is that same vivacity from their neighbours, advocates, and wider community members to finally address the ongoing emergencies of toxic drugs and inadequate housing.

### Trustworthiness

To ensure credibility of the findings of my research, I engaged members of my committee and other members of my research team including students to help me conduct a member-checking exercise at a critical midpoint in my analysis [37,38]. Member-checking allowed me to gauge how accurately I was interpreting the participants’ meaning in their stories. To approach authenticity in my sample, I tried my best to recruit different voices through a purposive sampling method. We reached saturation and captured different perspectives from men and women, and from residents of Prince George and Vancouver. I am confident in the integrity of the work presented in this study; I take a self-critical, reflexive, relational, and collaborative approach to my work and was honest with myself, participants, and coauthors throughout every stage of this research. This looked like reflexive practice through journaling and checking in with participants during the interview; sharing who I am and where I come from over snacks, coffee or tea with participants; practicing mindfulness and active listening, both during interviewing and while conducting the analyses; and spending unstructured time at the Cedar study sites supporting whatever work was being done to ensure a safe and comfortable environment for participants.

## Limitations

A common critique of member-checking is that it attempts to validate emerging findings from the data by seeking confirmation from participants about ideas that emerged from their interviews. This can hinder analysis by masking alternative or unexplored or unexpected relationships within the data, or it can “derail you from good analytic interpretations” [25] if they disagree with what you have presented to them when you are on a good path. To help mitigate this, I only presented emerging themes I was already confident about, and primarily sought help with the wording and interpretation of those themes; and I conducted the member-checking exercise approximately halfway through coding the transcripts so that participants were able to *guide* some of the analysis, as active participants in the study. In the first iterations of my themes, I tried to maintain a strengths-based position to a fault, which resulted in glossing over hard truths and trauma experienced by participants. In one member-checking meeting, a participant said, “it’s okay to call it how it is: COVID-19 sucked, and it’s okay to say that.” This lesson reminded me to return to my relational approach and commitment to understand the *meaning* of participants’ stories, in *all* their emotional states.

I experienced a few challenges eliciting rich responses to some questions, particularly around safe drug supply: as a new researcher and one who was not involved in the day-to-day operations at the Cedar Project, I may not have had enough rapport as an interviewer to elicit richer answers from the participants I interviewed. I have more to learn about relationship building with my kin in research contexts, particularly about subjects in which I do not have lived experience and accept this limitation of my work. Similarly, I cannot discount bias from both myself as researcher, and participants in this study. Social desirability bias from participants may have led them to overestimate their engagement with COVID-19 restrictions and protective measures.

## Conclusion

The stories shared in this study demonstrate the profound impact of historical and ongoing inequities on urban IPWUD during the COVID-19 pandemic. We found that pre-existing sociodemographic disparities such as unstable housing and substance use were exacerbated by the crisis, influencing perceptions of COVID-19 risk, access to essential services, and community dynamics. The interconnectedness of these challenges highlighted the critical need for equitable distribution of resources and support systems during critical moments in an emergency, especially for IPWUD. By recognizing and addressing these inequities, both in the immediate response to crises like COVID-19 and in broader systemic reform, we can better support the resilience and well-being of IPWUD. It is imperative that health authorities continue to develop inclusive and equitable public health strategies that prioritize the voices and needs of those most affected by intersecting crises.
